# Intra-operative assessment of the vascularisation of a cross section of the meniscus using near-infrared fluorescence imaging

**DOI:** 10.1007/s00167-021-06690-w

**Published:** 2021-08-04

**Authors:** Peter van Schie, Thies J. N. van der Lelij, Maxime Gerritsen, Ruben P. J. Meijer, Ewoud R. A. van Arkel, Marta Fiocco, Jan-Willem A. Swen, Alexander L. Vahrmeijer, Hans Marten Hazelbag, Stijn Keereweer, Pieter B. A. A. van Driel

**Affiliations:** 1 Department of Orthopaedic Surgery, Leiden University Medical Centre, Albinusdreef 2, 2333 ZA Leiden, The Netherlands; 2 Department of Surgery, Leiden University Medical Centre, Leiden, The Netherlands; 3 Centre for Human Drug Research, Leiden, The Netherlands; 4 Department of Orthopaedic Surgery, Haaglanden Medical Centre, The Hague, The Netherlands; 5 Mathematical Institute Leiden University, Leiden, The Netherlands; 6 Department of Biomedical Data Science, Medical Statistics Section, Leiden University Medical Centre, Leiden, The Netherlands; 7 Department of Pathology, Haaglanden Medical Centre, The Hague, The Netherlands; 8 Department of Otorhinolaryngology Head and Neck Surgery, Erasmus Medical Centre, Rotterdam, The Netherlands; 9 Department of Orthopaedic Surgery, Isala Medical Centre, Zwolle, The Netherlands

**Keywords:** Meniscal vascularisation, Near-infrared fluorescence imaging, Indocyanine green, Intraoperative imaging, Total Knee Arthroplasty

## Abstract

**Purpose:**

The purpose of this study was to assess whether the vascularisation of the meniscus could be visualised intra-operatively using near-infrared fluorescence (NIRF) imaging with indocyanine green (ICG) in patients undergoing total knee arthroplasty (TKA).

**Methods:**

The anterior horn (i.e., Cooper classification: zones C and D) of the meniscus that was least affected (i.e., least degenerative) was removed during TKA surgery in ten patients to obtain a cross section of the inside of the meniscus. Thereafter, 10 mg of ICG was injected intravenously, and vascularisation of the cross section of the meniscus was assessed using the Quest spectrum NIRF camera system. We calculated the percentage of patients in whom vascularisation was observed intra-operatively using NIRF imaging compared to immunohistochemistry.

**Results:**

Meniscal vascularisation using NIRF imaging was observed in six out of eight (75%) patients in whom vascularisation was demonstrated with immunohistochemistry. The median extent of vascularisation was 13% (interquartile range (IQR) 3–28%) using NIRF imaging and 15% (IQR 11–23%) using immunohistochemistry.

**Conclusion:**

This study shows the potential of NIRF imaging to visualise vascularisation of the meniscus, as vascularisation was observed in six out of eight patients with histologically proven meniscal vascularisation.

**Level of evidence:**

IV.

## Introduction

Traumatic ruptures mostly occur in young and athletic people with non-degenerative meniscal tissue who benefit from surgical repair [10]. However, despite careful patient selection, improved surgical techniques and post-operative rehabilitation, failure rates of up to 39% have been reported [11, 12, 14]. The success rate can be improved by repairing only meniscal tears that are located in vascularised tissue, as the healing capacity of the meniscus is directly related to the blood supply [2, 22].

**Fig. 1:**
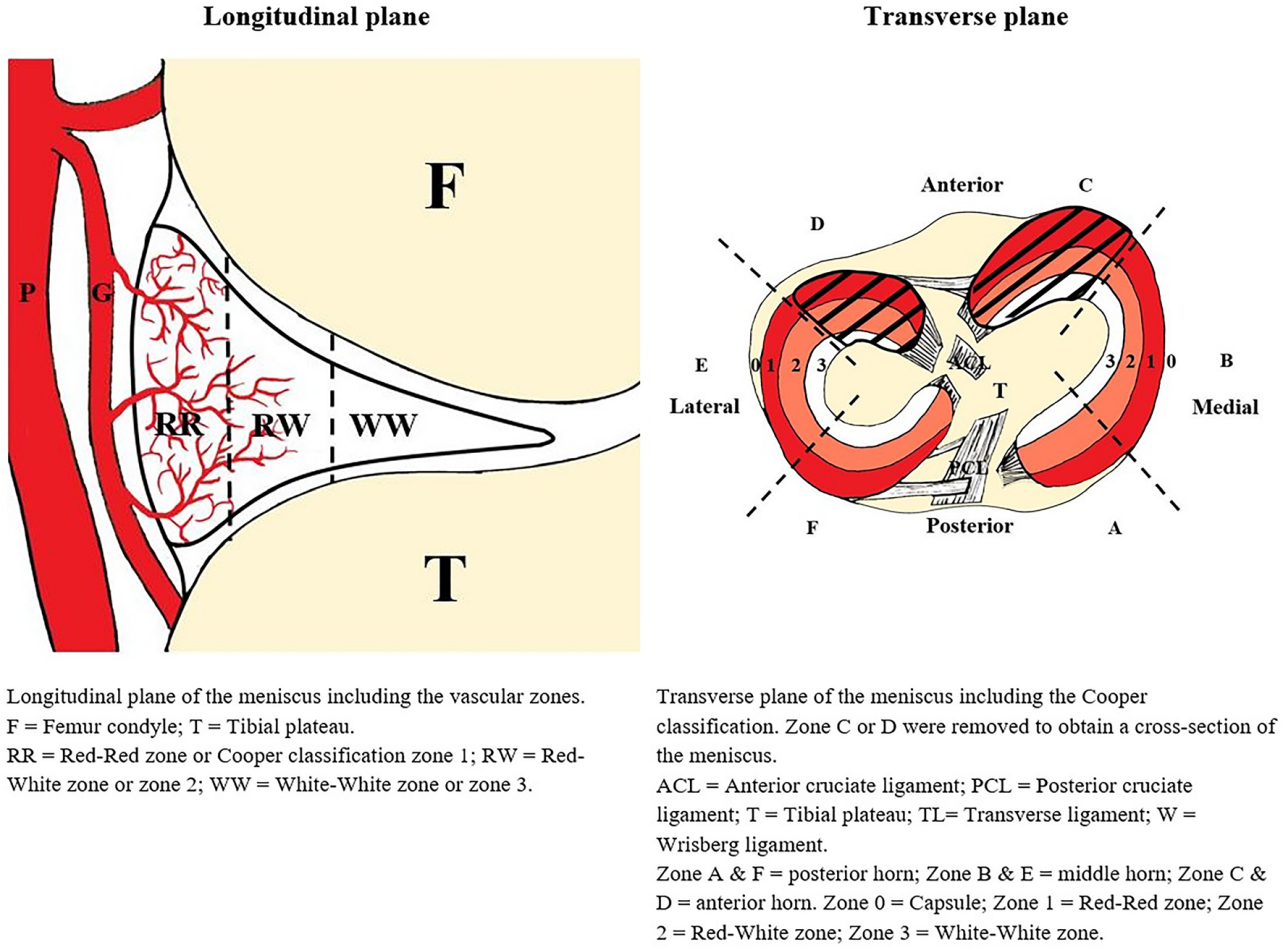
Schematic drawing of the human meniscus demonstrating the different vascular zone

The entire meniscus is vascularised at a young age, but in adults, blood vessels are only observed in the peripheral one-third of the meniscus [4]. Traditionally, the meniscus is divided into three zones to indicate the extent of vascularisation (Fig. 1). The outermost or peripheral zone, which is penetrated by vessels from the peri-meniscal plexus, is known as the vascularised red–red zone or zone 1. The most centrally located zone is known as the avascular white–white zone or zone 3. Zone 2 is located between the red–red and white–white zones and is known as the transition zone or red–white zone, where blood vessels may occasionally be present. Vascularisation is expected to play a major role in healing after repair, as a meniscal tear located in the most vascularised red–red zone has a higher chance of healing than a tear in another zone [21, 22]. However, large variation among individuals in the extent of vascularisation has been reported, making it difficult to assess pre- and intra-operatively whether a tear is located in vascularised tissue [2, 7].

**Fig. 2:**
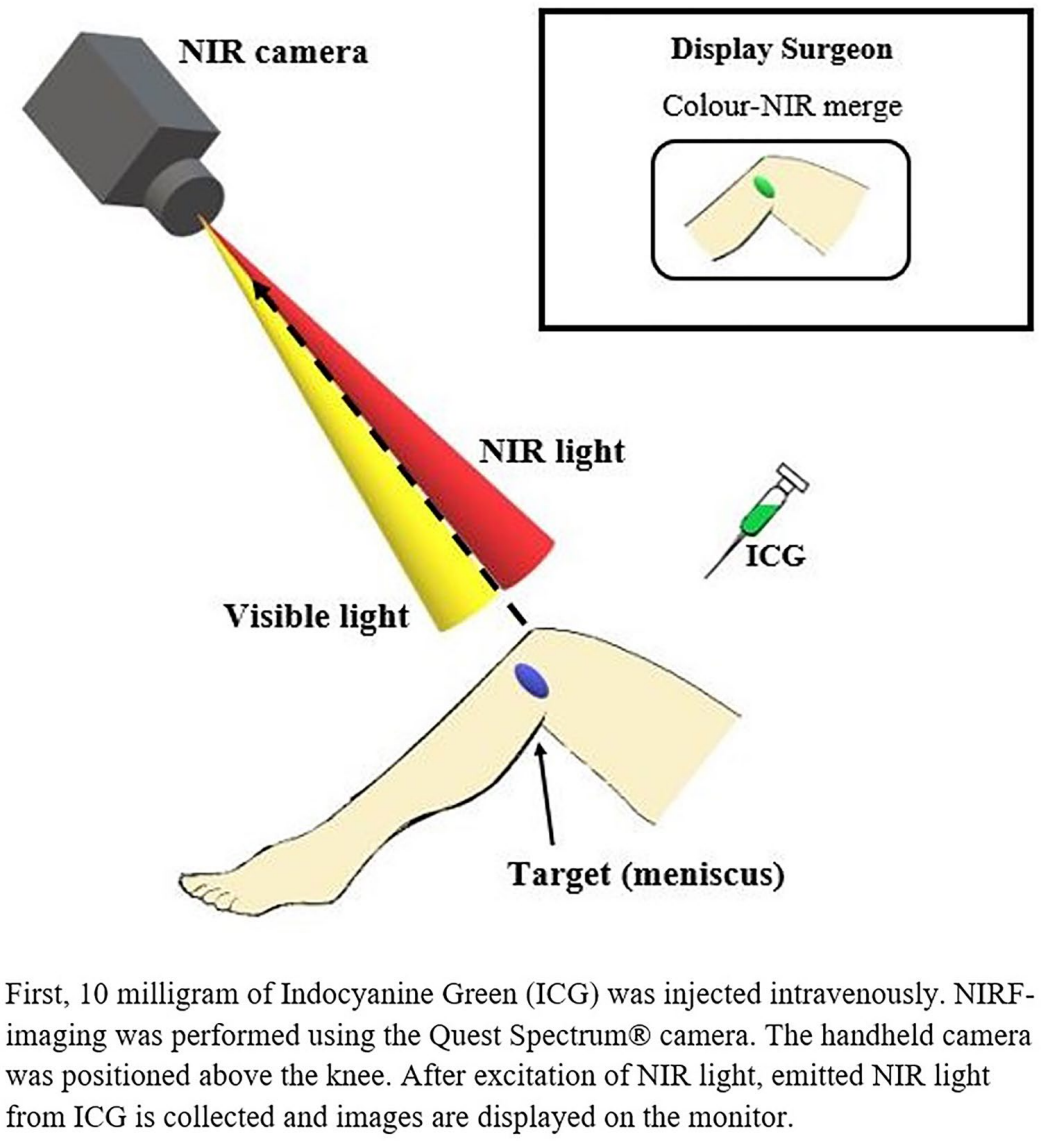
Overview of the NIRF-imaging setup

Near-infrared fluorescence (NIRF) imaging is an emerging and promising tool for the intra-operative visualization of (micro-)vascularisation and could play a role in assessing whether a meniscal tear is localised in vascularised tissue [15–18, 20]. It uses a near-infrared (NIR) camera system in combination with systemically injected NIR fluorophores, such as indocyanine green (ICG) (Fig. 2) [1]. In fluorescence imaging, fluorescent light is sent into tissue by the light source of the camera system. After absorption of the fluorescent light by the NIR fluorophores, the molecules reach an excited state (i.e., higher energy level). Subsequent return to the ground state results in the emission of fluorescent light of a higher wavelength that is detected by the camera system [23]. Imaging in the NIR region offers specific optical advantages (e.g., low absorption and low tissue autofluorescence), resulting in relatively high tissue penetration. NIR light, with wavelengths between 700 and 900 nm, is invisible to the human eye and therefore does not interfere with the surgical working field [9]. The success of NIRF imaging of the micro-vascularisation of the meniscus is largely dependent on two factors: the optical properties of the meniscal tissue and the amount of ICG that penetrates the microvasculature of the meniscus. The optical properties of the meniscal tissue determine the light attenuation and consequently influence the detection of ICG [9]. Furthermore, every NIRF camera system has a detection threshold [19]. If NIRF imaging can be used intra-operatively to assess whether a meniscal tear is localised in vascularised tissue, it could guide intra-operative decision-making, e.g., toward repair or partial meniscectomy if the tear is localised in vascularised tissue or fully located in avascular tissue, respectively.

The aim of this study was to assess whether NIRF imaging with ICG can be used to visualise the vascularisation of the meniscus in patients undergoing total knee arthroplasty (TKA). This is the first study to evaluate the feasibility of intra-operative NIRF imaging to visualise the vascularisation of the meniscus. The hypothesis was that meniscal vascularisation could be observed using NIRF imaging and that there would be a positive correlation between the extent of vascularisation measured by NIRF imaging and immunohistochemistry. The percentage of patients in whom vascularisation of the meniscus was observed intra-operatively using NIRF imaging when detected with immunohistochemistry was reported.

## Materials and methods

The LUMC Medical Ethical Committee gave permission to conduct this study under Dutch law (CME, P17.267), and informed consent was obtained from all patients. This was a phase II single-centre, single-arm study to evaluate the feasibility of NIRF imaging in combination with ICG to visualise the vascularisation of the meniscus during surgery in TKA patients.

### Patients

10 consecutive adult patients with symptomatic osteoarthritis scheduled for TKA between August 2019 and September 2019 were included. All patients underwent a standard diagnostic work-up, including preoperative anteroposterior and lateral radiographs. MRI for pre-operative assessment of the meniscal status was not performed. The exclusion criteria were prior meniscectomy, prior meniscal tear repair, pregnancy, renal insufficiency, thyroid abnormalities or ICG/iodine/shellfish allergy. No patients were excluded because of the exclusion criteria. Seven patients underwent surgery on their left knee, and three underwent surgery on their right knee. The lateral meniscus was assessed in eight patients, and the medial meniscus was assessed in two patients. TKA was performed by three orthopaedic surgeons (PvD, EvA and J-WS).

### Surgical procedure

TKA was performed with the patient in the supine position. No tourniquet was used to ensure blood supply to the meniscus during ICG injection. After creating an anterior incision, a medial parapatellar arthrotomy was performed, the anterior cruciate ligament and Hoffa’s fat pad were resected, and the patella was everted. Bleeding from outside the area of interest was carefully stopped by coagulation. Thereafter, the anterior horn of the meniscus (Cooper classification: zone C or D) that was least affected by osteoarthritis (i.e., the least degenerative) was removed [5]. An incision was made from the capsule of the meniscus (Cooper classification: zone 0) up to and including the most centrally located part of the meniscus (Cooper classification: zone 3) to simulate a meniscal tear and obtain a cross section of the inside of the meniscus (Fig. 1). The knee was then covered with light-absorbing surgical drapes, leaving only the remaining meniscus visible. Any blood that could interfere with NIRF imaging analysis was regularly rinsed and dried. Simultaneously, the NIRF camera (Quest Spectrum^®^) was draped with a sterile sheet and positioned above the knee by one of the researchers (PvS and PvD). The camera was positioned by hand and could be moved freely. The NIRF camera setup is shown in Fig. 2. Subsequently, 25 mg of ICG (Verdye^®^, powder for solution for injection) was diluted with 10 ml of sterile physiological water. 4 ml of this suspension (i.e., 10 mg of ICG) was injected intravenously. NIRF imaging of the meniscal tear was then performed up to 10 min after ICG injection. If a fluorescent spot was observed in the meniscus, a superficial scratch was made with a scalpel at the most centrally location of the observed vascularisation. Thereafter, the meniscus, including a rim of capsular tissue, was removed, and the superficial scratch was marked with a suture (Ethilon^®^ 5.0). The menisci were stored in formaldehyde, and the NIRF images were saved. During the surgical procedure and video analysis, two researchers (PvS or PvD) searched for bleeding sites that were imaged by NIRF but could not be detected by the human eye. The presence of intravascular ICG or bleeding sites in the capsule of the meniscus indicated the presence of blood vessels in the capsule and the arrival of ICG in the meniscal environment. Finally, the tourniquet was inflated, and the TKA procedure continued as usual.

### NIRF camera (Quest Spectrum^®^ fluorescence imaging system)

The Quest Spectrum^®^ (Quest Medical Imaging, Middenmeer, The Netherlands) system was designed and developed for open and minimally invasive image-guided surgery using NIRF. The fluorescence imaging system was designed to visualise ICG not visible to the naked eye. During imaging, a colour image of the surgical field is presented simultaneously with the NIRF image to allow surgical guidance. Furthermore, two overlay screens can be used, including a fluorescence intensity map and a NIRF image that is projected over the coloured image.

### Video analysis

**Fig. 3:**
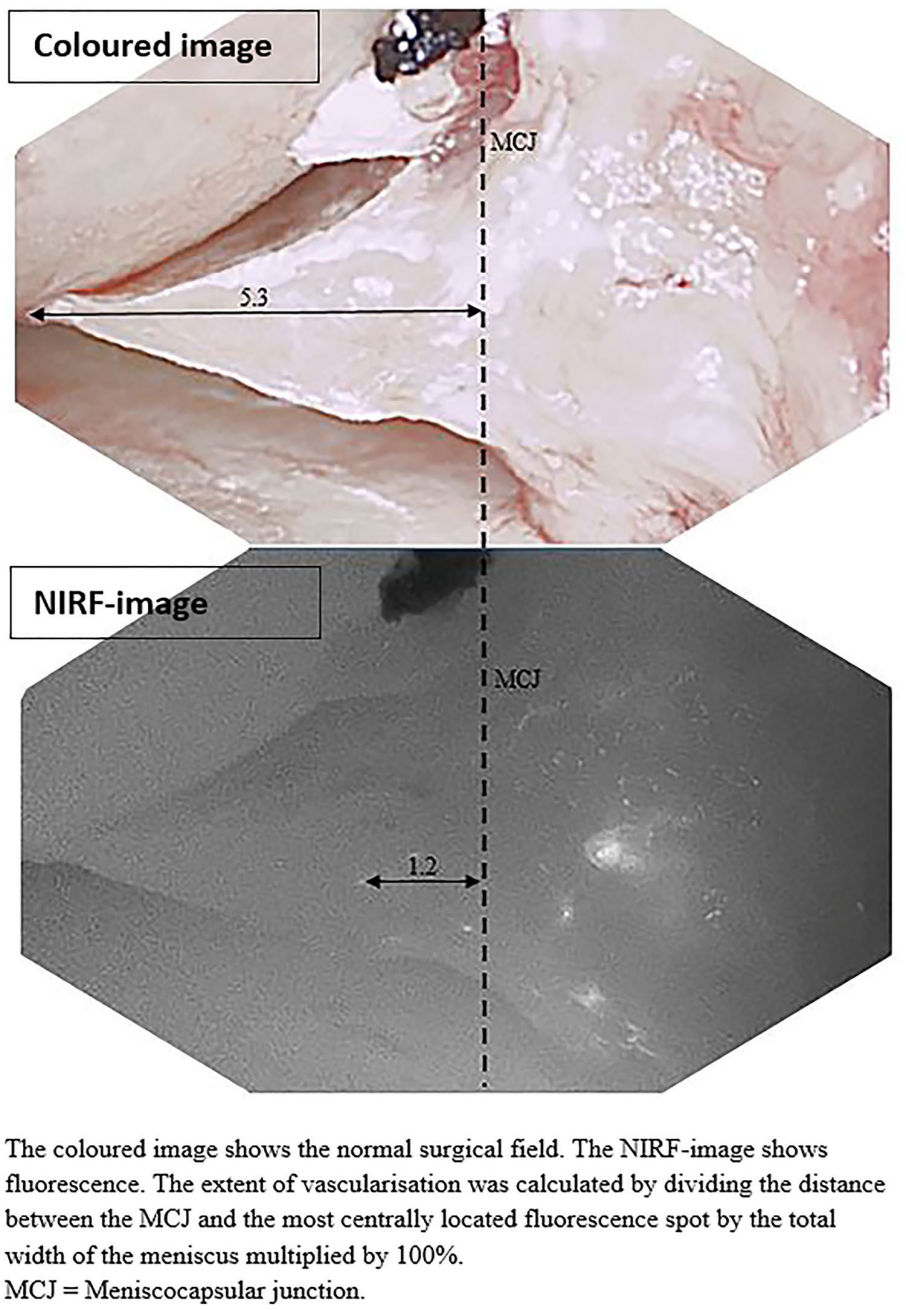
Measurements on intra-operative images of the meniscus

The procedure was recorded using the NIRF camera system, and images were recorded for 10 min after ICG injection. The NIRF images were post-analysed to assess whether meniscal vascularisation was visible in the 10 min after ICG injection. NIRF imaging was performed on the part of the meniscus that was least affected by osteoarthritis. The extent of vascularisation was calculated by dividing the distance between the menisco-capsular junction (MCJ) (Cooper classification: boundary between zones 0 and 1) and the most centrally located fluorescence spot by the total width of the meniscus measured as the distance between the MCJ and the most centrally located end of the meniscus (Cooper classification: up to and including zone 3) multiplied by 100% (Fig. 3). This means that in the case of a measured vascularisation of 13%, the vascularisation was limited to the peripheral third (Cooper classification: zone 1), while in the case of 39%, the vascularisation extended to the middle third (Cooper classification: zone 2). Measurements were performed using the Quest Research Framework (Quest Medical Imaging, Middenmeer, The Netherlands).

### Immunohistochemistry

The portion of the anterior horn (Cooper classification: zone C or D) adjacent to the middle horn (Cooper classification: zone B or E, respectively) was sectioned and stained with standard haematoxylin and eosin (H&E) and CD31 (clone JC70A, 1:200, Dako, Agilent, Glostrup, Denmark). This portion of the anterior horn was analysed, as it was directly adjacent to the cross section of the meniscus where NIRF images were obtained. CD31 is a specific marker for endothelial cells and therefore can be used to visualise even the smallest vessels. CD31-stained slides were used to measure the extent of vascularisation. The extent of vascularisation was measured and calculated in the same way as for the NIRF images.

### Statistical analysis

This was an exploratory study including ten participants, a number often used in explorative human studies.

The primary outcome was computed by dividing the number of menisci with vascularisation observed using NIRF imaging by the number of menisci with vascularisation observed by immunohistochemistry. This fraction was then multiplied by 100%.

As a secondary outcome, the correlation coefficient between the extent of vascularisation measured with NIRF imaging and immunohistochemistry was estimated. This study only included ten patients and was not intended to find a correlation. The Pearson correlation was used. The strength of positive and negative correlations was defined as follows: ≤ 0.35, weak; > 0.35–0.67, moderate; and > 0.67–1, strong [13]. The analyses were performed using SPSS version 25 (IBM Corp, USA).

## Results

### NIRF imaging

**Fig. 4:**
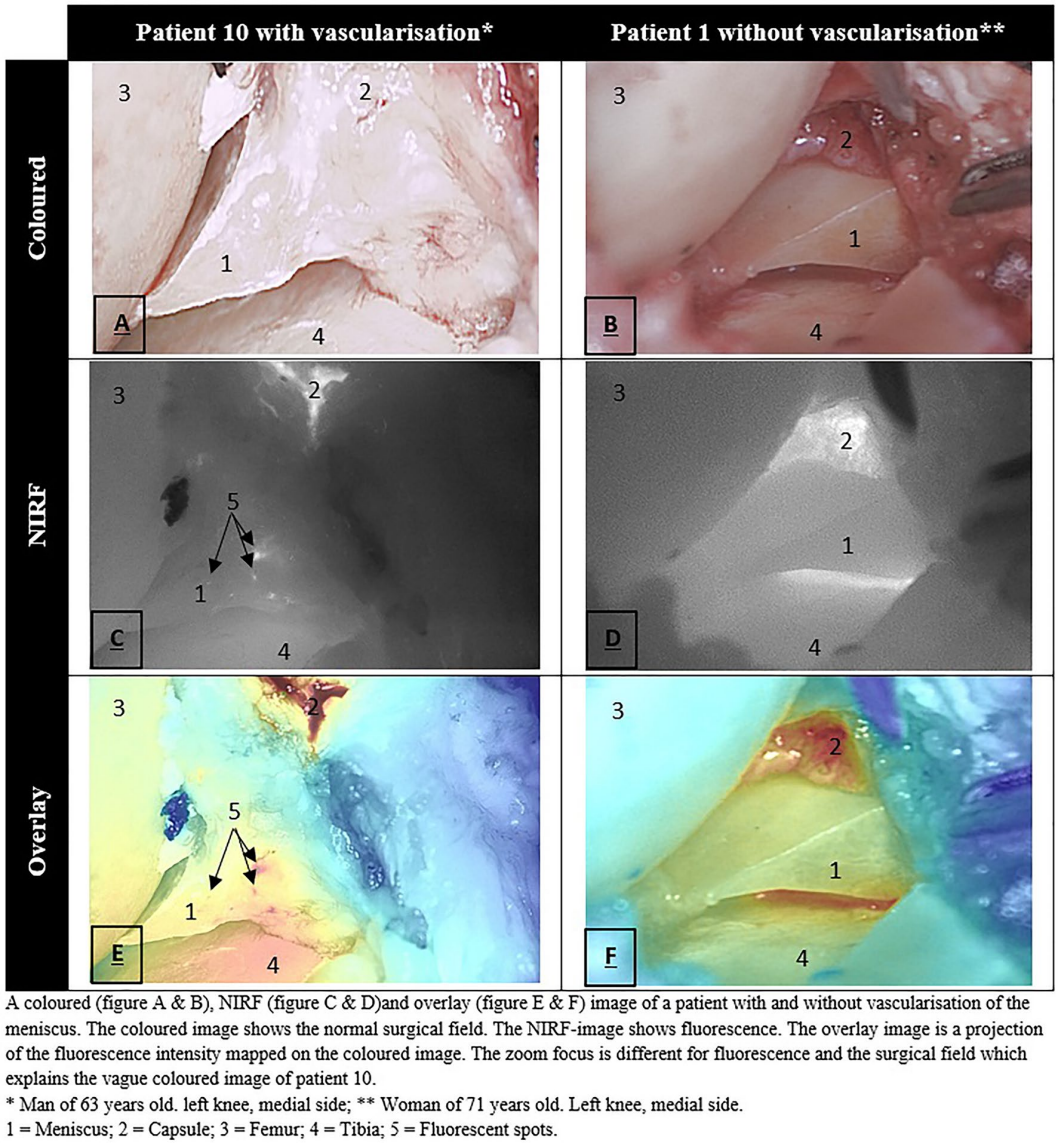
Intra-operative imaging

Patient characteristics are shown in Table 1. For two patients, the NIRF camera was not properly focused during surgery due to incorrect camera settings, resulting in poor-quality (blurry) images that could not be used for analysis. In the remaining eight patients, vascularisation was observed in six patients using NIRF imaging. In all of these six patients, vascularisation was detected with immunohistochemistry (Table 2). The median extent of meniscal vascularisation measured with NIRF imaging was 13% (interquartile range (IQR) 3–28%). Vascularisation of the knee capsule was clearly observed in all eight patients within 2 min of ICG injection, and as expected, a significant decrease in fluorescence was observed from the joint capsule towards the central part of the meniscus. In addition, we observed the presence of meniscal vascularisation due to spot bleeding in all patients. Importantly, bleeding sites and vascularisation visualised with NIRF imaging could not be seen by the human eye. An example of fluorescent spots is shown in Fig. 4C. A video of the full NIRF imaging procedure is included as supplementary material.

**Table 1:** Patient characteristics

	Frequency
Sex (male)	6
Smoking	3
Hypertension	2
Diabetes mellitus	1
Cardiovascular disease	0
ASA classification	
ASA I	3
ASA II	7
Kellgren–Lawrence classification*	
II	1
III	2
IV	7

	Mean (SD)
Age (years)	64 (8)
BMI (kg/m^2^)	29 (4)

*BMI* body mass index, *ASA* American Society of Anesthesiologists, *SD* standard deviation

*The classification was applied to the least osteoarthritic side

**Table 2:** NIRF imaging and immunohistochemistry

	Patients (*n* = 10)
Vascularisation detected (yes/no)	
Intra-operative NIRF imaging	
Yes	6
No	2
N/A	2
Immunohistochemistry	
Yes	9
No	1
N/A	0
Part of meniscus vascularized (%)	
Intra-operative NIRF imaging	
Median (IQR)	13 (IQR 3–28)
Range	0–39
Immunohistochemistry	
Median (IQR)	15 (11–23)
Range	0–47

*IQR* interquartile range, *NIRF* near-infrared fluorescence, *N/A* not applicable, because the images were not well focused

### Immunohistochemistry

**Fig. 5:**
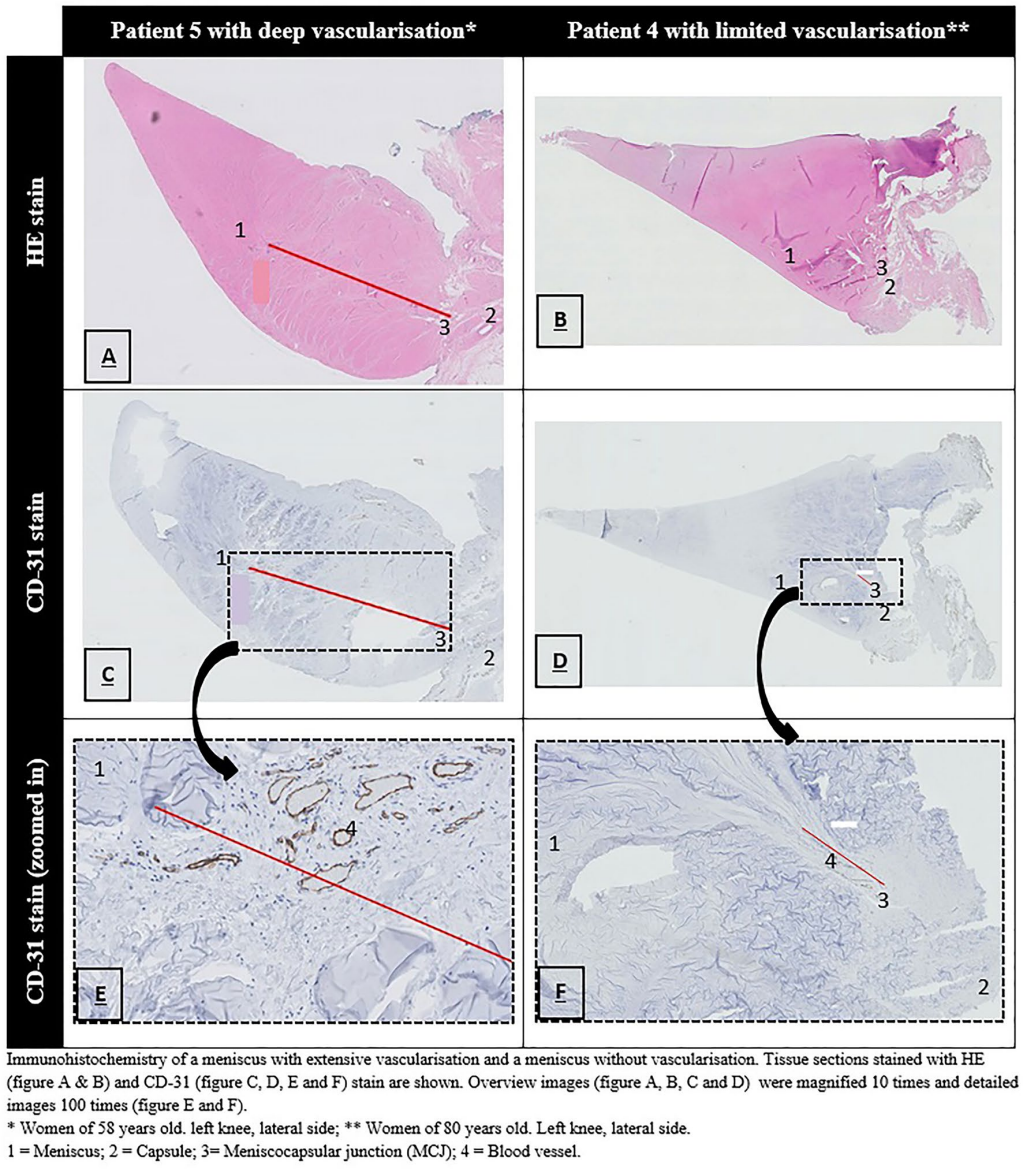
Immunohistochemistry of the meniscus

Blood vessels were observed in tissue sections from 9 patients using H&E and CD31 staining (Table 2). The median extent of meniscal vascularisation was 15% (IQR 11–23%). Vascularisation ran to the peripheral one-third in seven patients and into the middle one-third in two patients. In one patient, no vascularisation was observed. In seven patients, the periphery of the meniscus was mainly supplied by blood vessels from the peri-meniscal capillary plexus of the capsule, and in two patients, the meniscus was covered caudally and cranially with vascular synovial tissue from which blood vessels penetrated the periphery of the meniscus. H&E- and CD31-stained tissue sections from a patient with extensive vascularisation and a patient with limited meniscal vascularisation are shown in Fig. 5.

### Correlations

No correlation was found regarding the extent of vascularisation measured using NIRF imaging and immunohistochemistry (*r* = − 0.15 [95% CI − 0.71 to 0.53]).

## Discussion

The most important finding of the present study was that vascularisation of the meniscus was observed in six out of eight patients using NIRF imaging. In all patients, vascularisation of the knee capsule was clearly visible. Moreover, bleeding sites that could not be seen with the human eye were visible with NIRF imaging. This study shows that NIRF imaging is able to identify meniscal vascularisation in open surgery and is the first step towards arthroscopic NIRF imaging of meniscal vascularisation to guide meniscal repair surgery. Further research is needed to explore the feasibility of NIRF imaging during arthroscopy.

Danzig et al. reported a mean extent of vascularisation of 20% measured in 25 human cadaver menisci, which was comparable to the median of 15% found in this study [7]. In contrast, Arnoczky et al. studied 20 menisci and reported vascular penetration ranging from 10 to 25% for the lateral meniscus and 10–30% for the medial meniscus, which is less than the 37% and 47% for two patients in this study (Table 2) [2]. This indicates that in some patients, the extent of meniscal vascularisation is greater than what would be expected from previous literature [2, 4, 7]. This indicates that in some patients a greater proportion of the meniscus is vascularised than what would be expected from previous literature. Following these histological results, more research is needed to assess the variation among individuals. The extent of vascularisation in the median and lateral meniscus could not be reliably compared, as only two medial menisci were included.

Further research is needed to investigate the feasibility of arthroscopic NIRF imaging during arthroscopic meniscal repair surgery. A large amount of research on laparoscopic NIRF imaging has already been performed, which paves the way for arthroscopic applications [3]. However, several challenges remain, as arthroscopic meniscal repair is usually performed using a tourniquet and with irrigation to improve intra-operative visualization. The inflow of ICG into the meniscal vessels is only possible with (temporary) release of the tourniquet, with the disadvantage of intra-articular blood accumulation during the operation, resulting in an NIRF signal that may obscure the NIRF signal of the blood vessels in the meniscus. Nevertheless, the arthroscopic camera can be positioned close to the meniscus, and bleeding vessels that distort the images can be coagulated. Furthermore, a tourniquet is not mandatory for an arthroscopic procedure, and certainly, the use of modern arthro-pumps enables good visualisation without the use of a tourniquet. Whether NIRF imaging could be used during dry and/or wet arthroscopy should be investigated. Doi et al. already performed NIRF imaging during arthroscopy of the shoulder, demonstrating that this should be possible [8]. In current practice, a meniscal tear is arthroscopically grated before a suture is placed, thereby inducing spot bleeding. Since spot bleeding may not be visible during wet arthroscopy, we expect dry arthroscopy to be preferred during NIRF imaging. If follow-up studies show that it is possible to distinguish between vascularised and non-vascularised meniscal tissue using arthroscopic NIRF imaging techniques, this may help surgeons make a decision intra-operatively between meniscal repair and partial meniscectomy.

To the best of our knowledge, this is the first study to investigate the feasibility of NIRF imaging for the intra-operative assessment of meniscal vascularisation. However, several limitations should be noted. First, a correlation was not found regarding the extent of vascularisation measured with NIRF imaging and immunohistochemistry. Although the histological sections were obtained from close to the NIRF imaging site, the locations where NIRF imaging and immunohistochemistry were performed may vary slightly and therefore result in incongruent measurements. This may also explain why the extent of vascularisation measured using NIRF imaging was greater than that measured by immunohistochemistry in three patients (Table 2). Second, no sample size calculation was performed prior to this study, as finding a correlation was not our primary outcome of interest. Third, measurements on NIRF images and immunohistochemistry were performed once by a single person (PvS). However, we expect that these single measurements have hardly affected the outcomes, as both researchers (PvS for NIRF imaging and H-MH for immunohistochemistry) were well trained to perform these measurements. Fourth, known limitations of NIRF imaging are scattering, absorption and autofluorescence, which may limit the ability to detect the intra-vascular NIR fluorescence signal and distinguish it from its surrounding tissue [9]. Fifth, the validity and generalizability of our results are limited, as this study includes elderly patients with osteoarthritis, a population that is different from the target population consisting mainly of young patients with a traumatic meniscal tear. Nevertheless, both the extent of vascularisation and the vascularisation density are expected to be higher in young adults than in elderly adults, suggesting that this technique should also be feasible in a younger population [6].

## Conclusion

The use of NIRF imaging to assess meniscal vascularisation is promising, as vascularisation was observed in 75% of patients with histologically proven meniscal vascularisation.
